# On-line monitoring of methanol and methyl formate in the exhaust gas of an industrial formaldehyde production plant by a mid-IR gas sensor based on tunable Fabry-Pérot filter technology

**DOI:** 10.1007/s00216-016-0040-9

**Published:** 2016-11-09

**Authors:** Andreas Genner, Christoph Gasser, Harald Moser, Johannes Ofner, Josef Schreiber, Bernhard Lendl

**Affiliations:** 1 Institute of Chemical Technologies and Analytics, TU Wien, Getreidemarkt 9/164, 1060 Vienna, Austria; 2 Metadynea Austria GmbH, Hafentrasse 77, 3500 Krems an der Donau, Austria

**Keywords:** Formaldehyde production, Fabry-Pérot detector, Mid-infrared, Process analytical chemistry, Methyl formate, Methanol

## Abstract

On-line monitoring of key chemicals in an industrial production plant ensures economic operation, guarantees the desired product quality, and provides additional in-depth information on the involved chemical processes. For that purpose, rapid, rugged, and flexible measurement systems at reasonable cost are required. Here, we present the application of a flexible mid-IR filtometer for industrial gas sensing. The developed prototype consists of a modulated thermal infrared source, a temperature-controlled gas cell for absorption measurement and an integrated device consisting of a Fabry-Pérot interferometer and a pyroelectric mid-IR detector. The prototype was calibrated in the research laboratory at TU Wien for measuring methanol and methyl formate in the concentration ranges from 660 to 4390 and 747 to 4610 ppmV. Subsequently, the prototype was transferred and installed at the project partner Metadynea Austria GmbH and linked to their Process Control System via a dedicated micro-controller and used for on-line monitoring of the process off-gas. Up to five process streams were sequentially monitored in a fully automated manner. The obtained readings for methanol and methyl formate concentrations provided useful information on the efficiency and correct functioning of the process plant. Of special interest for industry is the now added capability to monitor the start-up phase and process irregularities with high time resolution (5 s).

## Introduction

In process analytical chemistry (PAC), there is clear focus on providing dedicated solutions to a given measurement problem. In this regard, emphasis is put on different parameters/features with respect to laboratory equipment. Depending on the installation, in PAC, a number of requirements have to be met. This can involve robustness against environmental conditions (e.g., humidity, vibration, chemical substances in the air), a simple user interface (soft- and hardware), avoiding sample preparation, autonomous operation, and the possibility to forward the gained measurement data to a control center (e.g., Modbus, OPC, 4-20 mA signal [1, 2]). Over the time, many analytical techniques were adopted, optimized, and successfully integrated in industrial processes. The range of different instrumental techniques that were brought on-line includes not only a broad variety of measurement principles such as conductivity-, pH-, and particle-sensors but also highly optimized gas chromatography systems, advanced mass spectrometers, and alike [3, 4]. However, if possible, simple and rugged, sensor-like solutions are the preferred way for efficient on-line monitoring with high time resolution.

A well suited measurement principle for analyzing process streams in the gas phase is infrared spectroscopy. Almost every gaseous analyte (except noble gases and homonuclear diatomic molecules) absorbs radiation in the mid-infrared region (4000–400 cm^−1^) and both, quantitative and qualitative, measurements are possible. Moreover, different instrumental realizations of mid-IR spectroscopy were developed over time, allowing customers to select the best suiting instrument [5].

Until today, the most generic and thus flexible technology for mid-IR-based gas measurements are Fourier transform infrared (FTIR) spectrometers [6]. Usually, they cover the whole mid-IR range and are capable of recording a full spectrum of the sample. The spectral resolution is typically 1–4 wavenumbers, but it can be reduced if a higher measurement frequency is required. Depending on the analytical problem to be solved either simple integration of characteristic absorption bands or application of chemometric approaches are the preferred modes of data analysis. Concerning applicability in the chemical industry, FTIR spectrometers are available from many different suppliers and in use for in-line as well as on-line monitoring of process gases. The downsides of this technology are, for example, high cost, limited temporal resolution, and, in some cases, the need for especially trained employees, especially when it comes to maintaining multivariate calibration models.

Another group of mid-IR-based analyzers make use of recent advances in laser technology in particular of quantum cascade lasers (QCLs) or intra-cavity lasers (ICLs) [7]. Using these lasers as light sources, concentrations down to the ppb-ppt concentration can be measured at high speed [8–10]. Moreover, it is possible to avoid moving parts, allowing the design of robust and compact instruments. However, their multi-analyte capabilities are still restricted due to the limited tuning range of the corresponding lasers ([11, 12]). An important current disadvantages of these mid-IR laser-based analyzers is their rather high cost.

Alternatively, filter-based mid-IR analyzers are a different, well-established group of mid-IR-based sensors that is characterized by less analytical power but with the advantage of low cost compared to FTIR-based analyzers. Here, a filter transmits infrared radiation only in the region where the analyte of interest is absorbing. These transmission windows can be rather wide (>20 cm^−1^ [13]) and cannot compete with the resolution of FTIR spectrometers. Therefore, they are only suited for rather simple applications such as quantifying CO_2_, CO, or ethylene in air [14–17]. In filter-based gas sensors, both absorption measurements based on Beers law as well as photoacoustic measurements have been realized so far.

If several analytes have to be quantified with the same analyzer, multiple filters with distinct transmission windows are needed. In the past, this was realized by mounting filters on a rotating filter wheel. However, the number of installable filters is generally limited, reducing somehow the possibility to fine tune across a certain spectral region as well as to select varying spectral segments with one and the same instrument.

Sensors which employ the gas filter correlation spectroscopy as measurement principle are closely related to the previously mentioned filter-based systems. Hereby, a gas cell filled with the analyte to be measured acts as the optical filter and generates the reference measurement [18, 19]. This technology is not limited to the infrared region ([20]) and typical analytes are CO, CO_2_, and SO_2_.

An approach for realizing filters is to use a Fabry-Pérot interferometer. Its basic principle is that two parallel and reflective surfaces allow only certain wavelengths to transmit. The transmitted wavelength segment depends on the distance between the reflecting mirrors (*d*), their reflectivity (*R*), and the interference order (*m*). The mathematical relation is as follows [21]:

$$ {\mathrm{FWHM}}_{\lambda }=\frac{2d}{\pi {m}^2}\frac{\left(1-R\right)}{\sqrt{R}} $$

Based on this technique, full widths at half height of typically 10–20 cm^−1^ can be achieved.

There are different ways how such FP filters have been implemented in process analyzers so far. FP filters with varying but mechanically fixed distances between the mirrors can be found in circular and linear variable filters [22]. Here, the first method is typically integrated in the respective instrument like a filter wheel, thus requiring a single detector, whereas instruments employing linear variable filters also contain a detector array. In these systems, the optical configuration is such that each detector element is irradiated by a different wavelength segment.

**Fig. 1:**
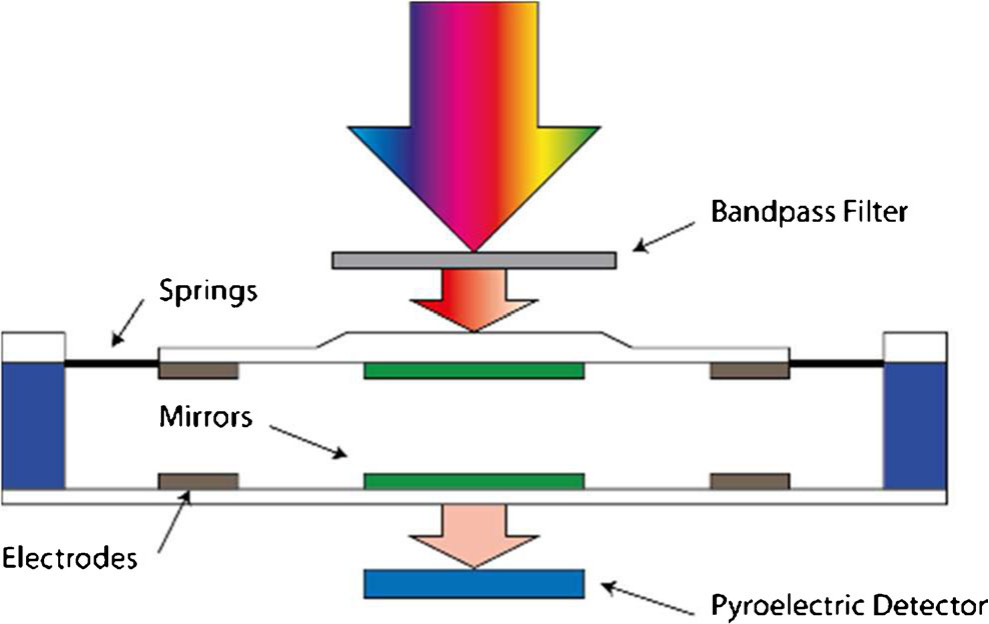
Scheme of a Fabry-Pérot filter-based detector

Applying microelectromechanical systems (MEMS) made it possible to develop Fabry-Pérot (FP) interferometers with variable distance between the reflective mirrors. Commercial available detectors employ either piezos (e.g., VTT Technical Research Centre of Finland Ltd. [23]) or mechanical springs (InfraTec GmbH) in combination with an electrical field to establish a certain distance between the mirrors and thus to select a certain wavelength segment. Realization of tunable FP filters using MEMS components allowed downsizing of this functional element. A sensor consisting of a tunable FP filter, a pyroelectric detector and corresponding preamplifier electronics can thus fit in a TO-8 can. Nevertheless, a broadband filter still must be installed to suppress the transmission of harmonics. A basic scheme of such a FP filter-based detector element is illustrated in Fig. 1. A detailed mathematical description of its operation basics is available in [21, 24–26].

## Production of formaldehyde

The measurement device presented in this paper was developed to monitor the concentration of side products from chemical reaction plants producing formaldehyde (FA). The underlying catalytic chemical reaction is the partial oxidation of methanol, leading to primarily formaldehyde. Two major processes which differ in the employed catalyst types are used to produce FA on an industrial scale. The first one, which is also known as Formox process, uses metal oxides (e.g., vanadium, molybdenum, or iron oxide) and is operated in the temperature region of 270–400 °C. The other one, which is also used at the investigated production plants of this study, is based on silver crystals and operated at significantly higher temperatures (600–720 °C) [27–30]. The formation of FA can be written as follows:

$$ {\mathrm{CH}}_3\mathrm{O}\mathrm{H}\ \leftrightarrow\ \mathrm{H}\mathrm{CHO} + {\mathrm{H}}_2\left(\Delta \mathrm{H} = +84\ \mathrm{kJ}/\mathrm{mol}\right) $$

And with oxidation of the hydrogen:

$$ {\mathrm{CH}}_3\mathrm{O}\mathrm{H} + 0.5\ {\mathrm{O}}_2\leftrightarrow \mathrm{H}\mathrm{CHO} + {\mathrm{H}}_2\mathrm{O}\ \left(\Delta \mathrm{H} = -159\ \mathrm{kJ}/\mathrm{mol}\right) $$

**Fig. 2:**
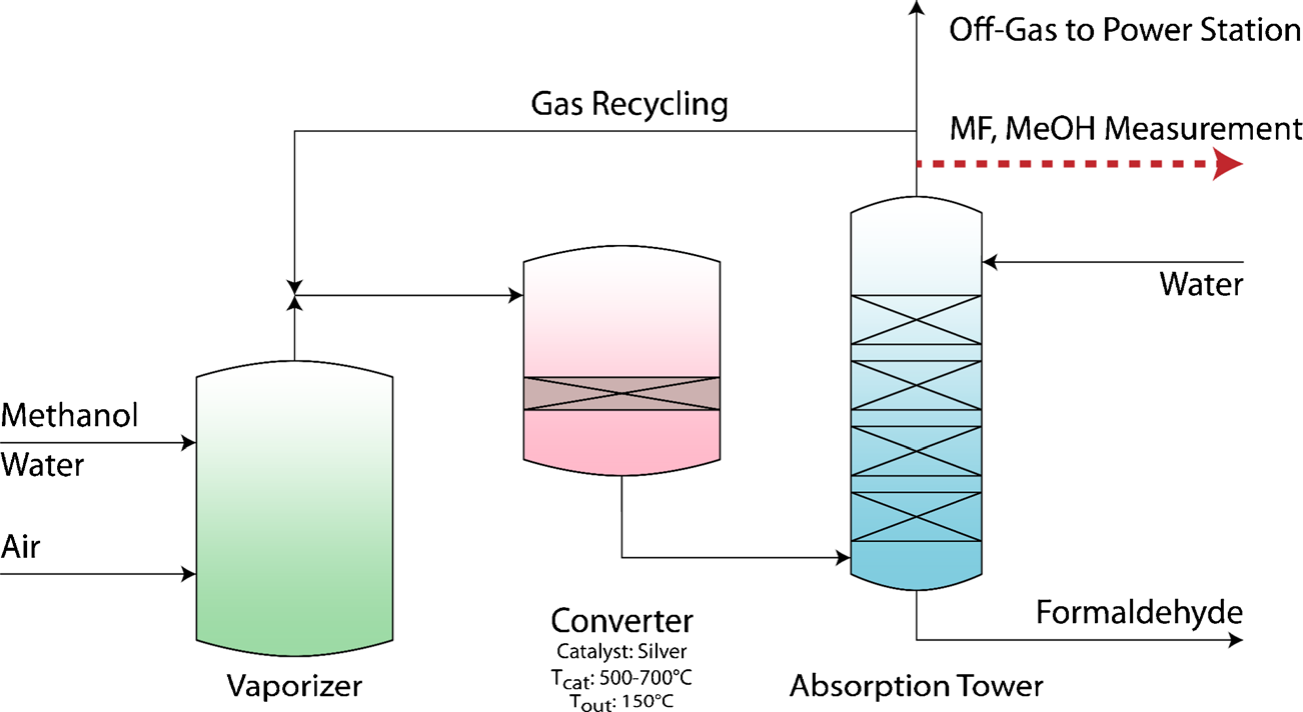
Simplified scheme of the FA production process based on the silver catalyst. The side product methyl formate (MF) and traces of not converted MeOH are quantified at the top of the absorption tower, indicated with a *red arrow* [31, 32]

After the catalytic reaction, the product stream is cooled down to approximately 150 °C and washed in counter flow with H_2_O in an absorption column (a simplified scheme is given in Fig. 2).

**Fig. 3:**
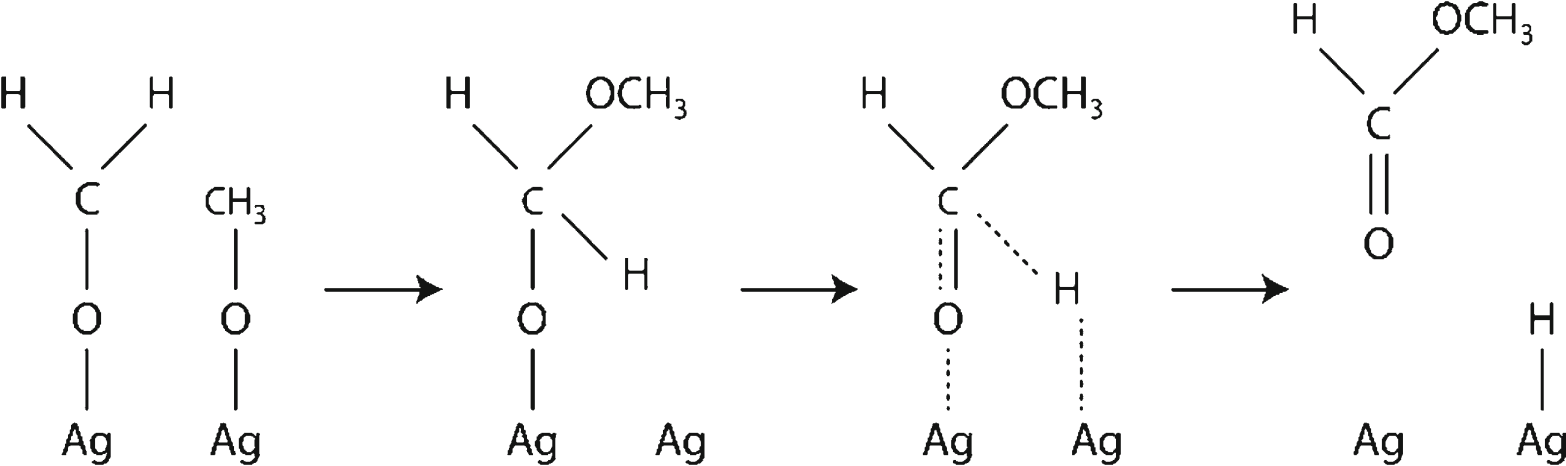
Reaction mechanism for the formation of methyl formate as proposed by Wachs and Madix

The main part of the off-gas consists of CO_2_, CO, and H_2_, which are already monitored at Metadynea Austria GmbH with commercial available devices. However, also low concentrations of methanol (MeOH) and methyl formate (MF) (both <5000 ppm) and traces of not absorbed FA (<50 ppm) can be detected. While MeOH origins from not converted reactant, MF is created by a side reaction on the silver catalyst. Investigations with deuterated methanol [33], performed at lower temperatures than in commercial processes, propose the mechanism shown in Fig. 3 (Tischenko mechanism).

However, Wachs and Madix mention that no MF is found in industrial processes. They argue that the catalyst temperature (>600 °C) would be too high to enable a long enough surface residence time of FA on the silver catalyst to react to MF.

The task of the newly developed mid-IR-based gas sensor is quantification of MeOH and MF in the process off-gas with high time resolution (5 s.). The sensor was developed and implemented with the vision to enable accurate monitoring of the chemical status of the process and therefore to open the possibility for a more economic operation of the FA production plants.

## Experimental setup

The installed mid-IR source is a JSIR350-4-AL-R-D6.0-0-0 (Micro Hybrid Electronic GmbH), which is a highly efficient blackbody emitter [34] and produced by applying MEMS processes. It is basically an electrical resistor which heats up when a voltage is applied. Due to its compact design and low thermal mass, amplitude modulation of the emitted radiation up in the hundred Hz region can be achieved. This allows to omit chopper wheels or other modulation techniques usually required by the need of the employed cost-effective pyroelectric detector. For this application, the applied voltage was 5 V and the modulation frequency was set to 3.5 Hz (duty cycle, 50 %) to achieve an optimum detector responsivity.

A ZnSe lens (*f* = 50 mm, ThorLabs Inc.) collimates the beam and a flat gold mirror reflects the radiation to a custom built gas cell. Its optical length is 30 cm and its steel body is heated up to 45 °C to avoid possible condensation from the humid off-gas on the cell walls. The limited space requires an additional reflection of the beam form a second plane mirror before it is focused (ZnSe, *f* = 50 mm) onto the detector.

**Fig. 4:**
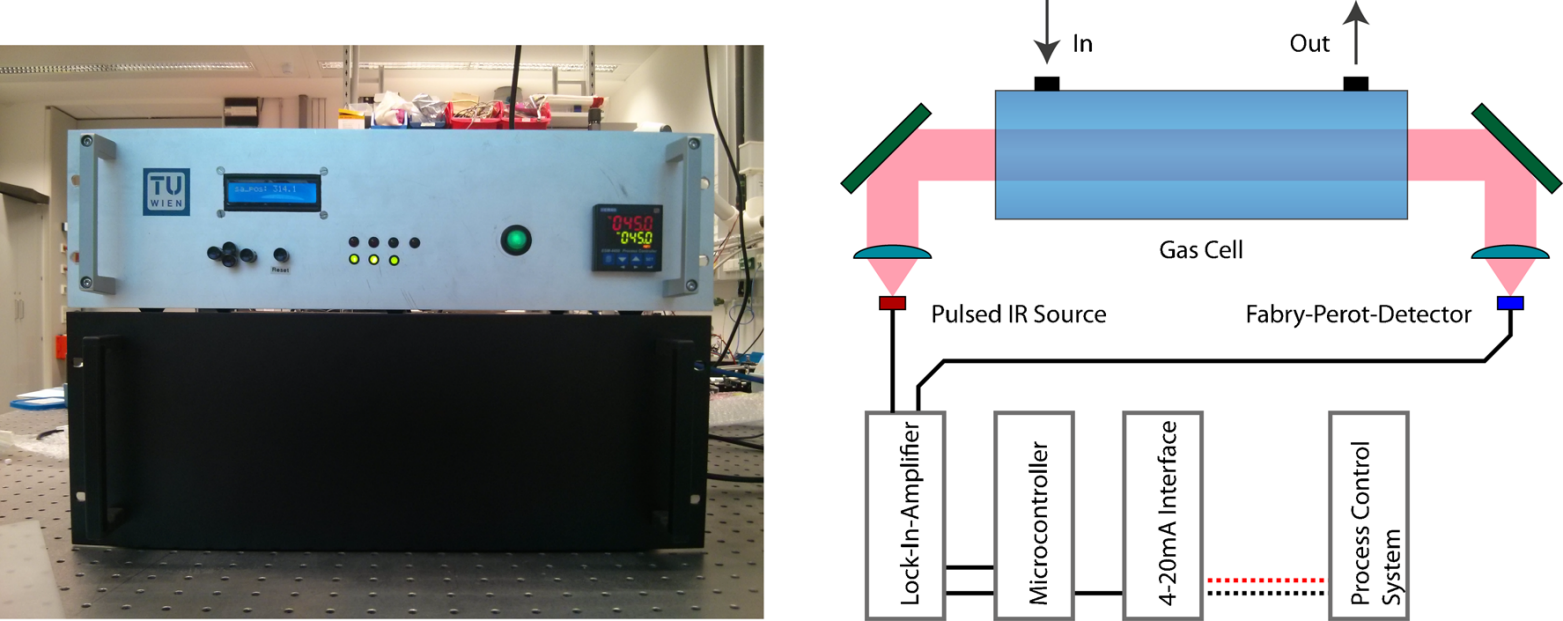
*Left*: front side of the developed sensor (19” rack compatible); *right*: schematic assembly of the optical and electrical parts

**Fig. 5:**
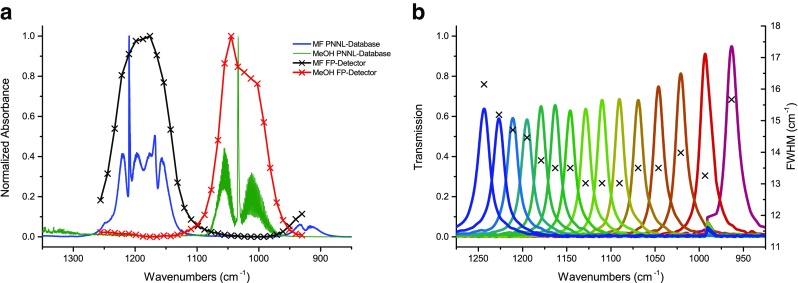
**a** Comparison of reference spectra (PNNL) and spectra recorded with the FP-detector. All spectra were normalized to a maximum absorbance of one. **b** Transmission behavior and FWHM of the Fabry-Pérot filter at different control voltages

The central component of the measurement device is the tunable Fabry-Pérot (FP) filter-detector LFP-80105-337 (InfraTec GmbH) [35]. By applying a control voltage (*V*_range_ = 0-70 V), the filter can be tuned through the region of 1250-950 cm^−1^, where two vibrational transitions of MF and MeOH can be found (Fig. 5). These bands (MF: CH_3_ rocking [36] at ∼1190 cm^−1^ and MeOH: C-O str. [37] at ∼1040 cm^−1^) are spectrally separated well enough for the tunable filter to resolve the bands, although the low spectral resolution of the tunable FP of approximately 10 cm^−1^ (Fig. 4).

As the mid-IR source is modulated, the detector signal has to be demodulated with an in-house developed Lock-In-Amplifier. The resulting signal is digitized with an analog digital converter (ADC, ADS1115, 16 bit, Texas Instruments Inc.) and a microcontroller (ATmega328P, Atmel Corporation) averages 100 measurement points to improve the signal to noise ratio. As the measurement principle is based on the absorption of light, one can apply the Beer-Lambert Law and calculate the concentration according to

$$ A\left(\lambda \right)= \log \left(\frac{I_{\lambda}^0}{I_{\lambda }}\right)=\varepsilon \left(\lambda \right) cl $$

where *A*(*λ*) is the absorbance, *I*_*λ*_^0^ is the intensity recorded from a reference measurement at a certain wavelength segment, *I*_*λ*_ is the intensity recorded from of the sample channel at a certain wavelength segment, *ε*(*λ*) is the observed decadic molar absorption coefficient at that wavelength segment, *c* is the concentration of the analyte, and *l* is the pathlength.

In order to calculate absorbance and the concentration of the target analyte, one needs to know values for *I*_*λ*_ and *I*_*λ*_^0^. Here, the reference value *I*_*λ*_^0^ is gained by flushing the gas cell with the IR inactive gas N_2_. This reference measurement, which is also helpful to compensate for long term drifts, is initiated by the microcontroller and performed every 2 h 45 min. The concentrations of the two target analytes have to be quantified consecutively which requires adjusting the filter position periodically. Therefore, a digital to analog converter (DAC, MCP4725, 12 bit, Microchip Technology Inc.) is installed and sets the control voltage of the FP filter-detector.

The concentrations are determined by applying a calibration curve and proportional voltage signals for each analyte are output on additional DACs (2xMCP4725). These analog signals are connected to two 4-20 mA converters (PXU-20.924/RS, Brodersen Controls A/S) to meet the requirements of the process control system (PCS) at Metadynea Austria GmbH. The 4–20 mA interface is the preferred way to monitor the concentration of the analytes of interest. However, an LCD display (HD44780, Adafruit Industries LLC) is also installed at the front panel of the sensor to check the functionality. An additional single-board-computer (Raspberry Pi 2 Model B, Raspberry Pi Foundation) and a mobile broadband modem (E3531, Huawei Co. Ltd.) allows remote monitoring and firmware upgrades of the microcontroller.

## Experimental

### Recording spectra of the analytes and calibration curves

Due to the conditions of the gas stream the prototype has to quantify MF and MeOH in the gas phase. At normal temperature and pressure, the analytes of interest are liquids with a significant vapor pressure (MeOH, 13.02 kPa; MF, 63.46 kPa). In order to characterize the device performance and to record calibration curves, gaseous reference samples with similar concentrations as to be expected at the intended application site had to be prepared in the laboratory. The physical properties of MF and MeOH make it difficult to prepare stable calibration gas mixtures of accurately known composition by means of static methods [38]. In addition, static calibration gas mixtures of the readily condensable gases and vapors of MF and MeOH cannot be maintained under a pressure near the saturation limit without the occurrence of condensation. Therefore, the saturation method according to ISO 6145-9:2009 was employed for preparing calibration mixtures of the analytes [39]. Following this standard a saturated gas stream is produced, where the concentration of the desired component can be calculated using pressure and temperature readings logged during the experiments. The resulting saturated gas stream was then further diluted to the appropriate concentration with N_2_ by employing mass flow controllers (MFCs, red-y smart, Vögtlin Instruments AG) and a static mixer. Finally, the sample stream was fed into the developed prototype.

Reference spectra of MeOH and MF were recorded with the prototype to establish calibration curves. To do so, the control voltage of the FP filter was increased to get one data point every 10 cm^−1^. This led to 31 points per spectrum, taking 2 min.

### Online measurements

Operating the prototype at Metadynea Austria GmbH involved a modification of the microcontroller firmware, compared to the reference measurements in the academic laboratory. Instead of recording full spectra with 31 data points, only two filter positions were selected. These were selected at the maximum absorption of the analytes and resulted in one concentration value for MF and MeOH every 5 s.

Multiple FA productions plants are located at the production site. As only one plant can be monitored at a time, the process control system switches the exhaust gas to the prototype automatically. It is intended to analyze each plant at least once per working shift. The result is that in normal operation mode, each plant is monitored for 1–2 h, depending on the number of active plants. This automatic gas stream cycle is overwritten if the plant operators modify process parameters or restart individual production plants.

## Results

### Spectra of analytes

Two typical spectra of MF and MeOH recorded with the prototype are compared with reference spectra from the PNNL database [40] and shown in Fig. 5a. One can clearly see that the resolution obtained with the FP-interferometer-based instrument cannot compete with an FTIR spectrometer. Nevertheless, the absorption bands of the analytes are sufficiently isolated which allows the application of the developed instrument.

### Calibration curves

Calibration samples were prepared with the gas mixing rig and spectra were acquired with the prototype. Due to the fact that only a single point in the spectrum is used for each analyte during operation at the production plants, wavelength segments with maxima at 1010 cm^−1^ for MeOH and 1160 cm^−1^ for MF were selected as spectral positions to establish the corresponding calibration curves. No significant cross sensitivities were found in the concentration ranges of practical interest.

**Fig. 6:**
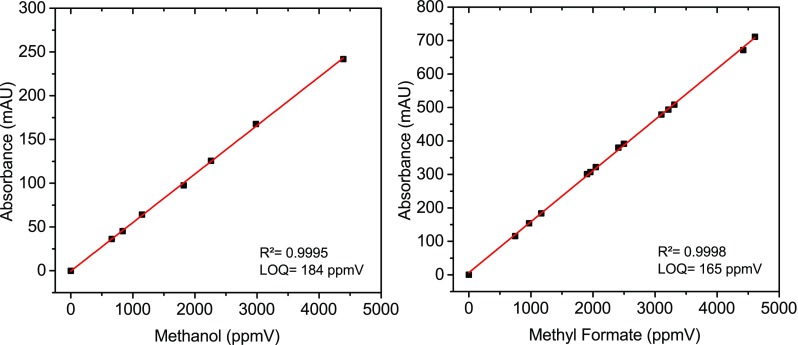
Calibration curves for MeOH and methyl formate, recorded at wavelength segments centered at 1010 and 1160 cm^−1^

The resulting calibration curves are plotted in Fig. 6, with achieved limits of quantification of 184 ppmV for MeOH and 165 ppmV for MF.

### Experiments at the production plants

Results from online-measurements at Metadynea Austria GmbH are depicted in the following figures. The exemplary data is typically plotted over several hours/several days. Due to company regulations absolute values, such as concentration values and production plant IDs (which also change during different experiments) and further additional plant parameters (catalyst temperature, etc.) may not be disclosed.

**Fig. 7:**
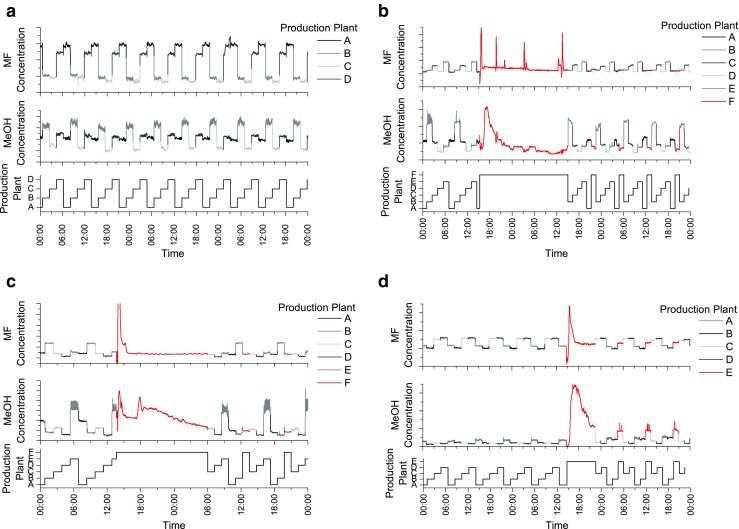
**a** Methly formate (MF) and MeOH concentration during 3 days at normal operation. **b**–**d** Retrieved concentration levels while starting an additional production plant (new plant indicated as *red sections*)

If the production parameters are constant, the data recorded from the PCS is as shown in Fig. 7a. Here, the periodical switching (approx. every 2 h) between four production plants initiated by the PCS can be observed. The constant production settings lead to almost stable MF and MeOH concentrations during 3 days of operation.

The FA production has to be stopped and restarted at certain intervals. The reasons for that are, for example, degradation of the catalyst caused by sintering effects [27] or test runs for other process optimization experiments. Three examples, where production plants have been restarted, are shown in Fig. 7b–d. During these processes, the automatic switching cycle was deactivated, to gain specific information on the selected reactor during these experiments. According to Wachs and Madix [33], MF can be produced on the silver catalyst at lower temperatures, which is the case when the FA production is started. Reaching the optimum production parameters also leads to a stable and relatively low MF concentration. The MeOH concentration does not stabilize as fast as MF which is very likely caused by its longer retention time in the absorption tower as a consequence of the higher water solubility of MeOH.

**Fig. 8:**
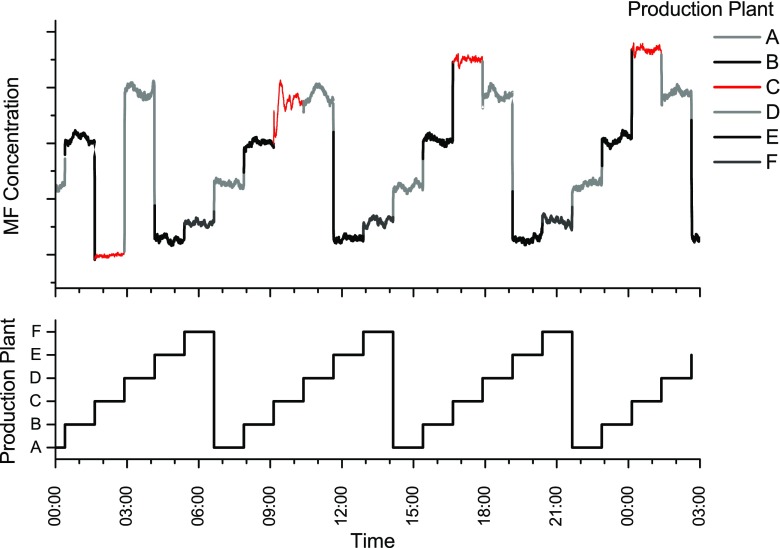
Redirecting the exhaust gas to the converter causes an increase of MF as the catalyst temperature decreases

A different experiment is shown in Fig. 8. Here, the exhaust-gas was redirected to the converter, leading to a decrease in temperature at the catalyst and an increase of MF at the measurement position. In this case, the automatic switching cycle was not deactivated and the new MF concentration was not accessible until the next repetition.

**Fig. 9:**
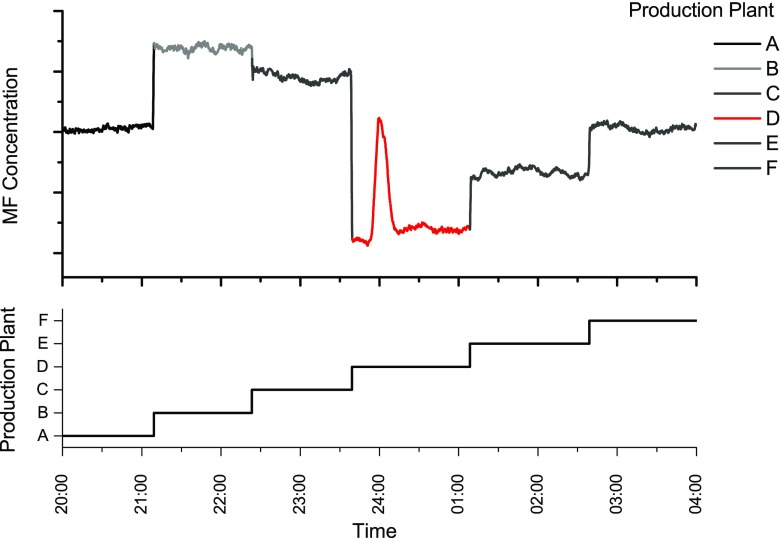
A short increase of the MF concentration due to a short change of catalyst temperature

Another example of the applicability of the developed process analyzer is shown in Fig. 9. An unexpected change of the catalyst temperature resulted in a quick increase of MF. The production parameters were reset within 15 min and the MF concentration stabilized immediately.

## Conclusion

A cost-efficient prototype of a process analyzer for on-line monitoring of MF and MeOH in the gas phase of a formaldehyde production plant was developed and implemented. Key components of the developed dedicated process spectrometer were an electrically modulated thermal IR source, a combined Fabry-Pérot interferometer-detector device and a microcontroller for automated measurements. A custom developed gas mixing rig allowed recording reference spectra and calibration curves of the analytes of interest. The achievable limits of quantification were 184 and 165 ppmV for MeOH and MF, respectively. The applicability of the prototype was shown at the production plants of Metadynea Austria GmbH. It provided valuable data on the time-dependent changes of the concentrations of the targeted process gases. After an initial installation phase, it is now considered as a valuable tool for monitoring the production plants and for providing in-depth information on the production process under investigation.
